# Seroprevalence of dengue virus infection in India

**DOI:** 10.6026/973206300222916

**Published:** 2026-05-31

**Authors:** Deepak Hindal, Gaurav Saxena, Shivani Sinha, Prafulla Songara, Mitisha Soni

**Affiliations:** 1 Department of Microbiology Government Medical College, Ratlam, Madhya Pradesh, India

**Keywords:** Dengue fever, Non-Structural protein 1 (NS1) antigen, seroprevalence, public health concern

## Abstract

Dengue fever has emerged as a significant public health concern in India, with increasing incidence rates observed across various regions. The NS1 antigen detection provides early and accurate diagnosis of dengue infection. Hence, a retrospective cross-sectional study was conducted from January 2024 to December 2024 at MC Ratlam (MCH), analysing 178 laboratory-confirmed dengue cases using NS1 Ag ELISA from a total of 1,898 tested samples. Demographic, clinical and geographical data were collected and analysed using appropriate statistical methods. The overall seroprevalence of dengue was 9.38% among tested samples. The mean age was 28.1 ± 15.2 years, with males comprising 63.5% of cases. The highest incidence was observed in the 21-30 years age group (29.2%).

## Background:

Dengue fever, caused by the dengue virus (DENV), has become one of the most rapidly spreading mosquito-borne diseases globally, with an estimated 390 million infections occurring annually[1]. The disease is endemic in over 100 countries, particularly in tropical and subtropical regions, where favourable climatic conditions support the breeding of Aedes mosquitoes [2]. Data indicates a steady increase in dengue cases over the past decade, with significant morbidity and mortality [3]. The state of Madhya Pradesh, located in central India, has witnessed periodic outbreaks of dengue fever, particularly in urban and semi-urban areas. The dengue virus belongs to the Flaviviridae family and comprises four distinct serotypes (DENV-1, DENV-2, DENV-3 and DENV-4) [4]. The clinical manifestations range from asymptomatic infections to severe dengue with plasma leakage, haemorrhage and organ involvement [5]. Early and accurate diagnosis is crucial for proper case management and prevention of severe complications. The NS1 (Non-Structural protein 1) antigen is a highly conserved glycoprotein secreted by infected cells during the early phase of dengue infection. NS1 antigen detection has emerged as a valuable diagnostic tool, particularly during the first week of illness when viral RNA levels are high [6, 7]. The NS1 Ag ELISA has demonstrated high sensitivity and specificity, making it an ideal screening tool for dengue diagnosis in resource-limited settings. Therefore, it is of interest to determine the seroprevalence of dengue virus infection by NS1 antigen detection in a tertiary care hospital in north-western Madhya Pradesh and analyse the associated epidemiological factors.

## Materials and Methods:

A retrospective cross-sectional study was conducted at MC Ratlam (MCH), a tertiary care hospital located in Ratlam district, north- western Madhya Pradesh, India. The hospital serves as a referral center for the surrounding districts and provides comprehensive healthcare services to both urban and rural populations. The study was conducted from January 2024 to December 2024, covering a complete calendar year that included pre-monsoon, monsoon and post-monsoon seasons. The study included all patients presenting with clinical features suggestive of dengue fever, such as acute onset of fever, headache, myalgia, arthralgia and other associated symptoms. Both inpatients (IPD) and outpatients (OPD) were included in the study. Patients with incomplete data, Patients tested by methods other than NS1 Ag ELISA and Duplicate entries were excluded. All serum samples were tested for dengue NS1 antigen using commercially available ELISA kits following manufacturer's instructions. The test was performed by trained laboratory technicians following standard operating procedures. A total of 1,898 samples were tested, of which 178 tested positive for NS1 antigen. Data was collected from laboratory records which included Demographic characteristics (age, gender and contact information), Geographic information (village/ward, sub-district, and district), Clinical presentation (OPD/IPD status, provisional diagnosis, date of onset), Laboratory parameters (test results, test dates) and Seasonal patterns and temporal distribution.

## Statistical analysis:

Data was analysed using Python programming language with appropriate statistical libraries. Descriptive statistics were calculated for continuous variables (mean, standard deviation, median, range) and categorical variables (frequencies, percentages). Chi-square tests were used to assess associations between categorical variables. A p-value of <0.05 was considered statistically significant.

## Ethical considerations:

The study was conducted following ethical guidelines for medical research. Patient confidentiality was maintained throughout the study period.

## Results:

A total of 1,898 samples were tested for dengue NS1 antigen, of which 178 tested positive, yielding an overall seroprevalence of 9.38%. All positive cases were confirmed by NS1 Ag ELISA and included in the final analysis. Among the 178 laboratory-confirmed dengue cases, males comprised 113 cases (63.5%) compared to 65 females (36.5%). The age distribution ranged from 7 months to 75 years, with a mean age of 28.1 ± 15.2 years and median age of 25 years as shown in Table 1. The temporal analysis revealed distinct seasonal patterns in dengue transmission. The highest number of cases was observed during the monsoon and post-monsoon months (August-November). October recorded the maximum number of cases (53 cases, 29.8%), followed by September (45 cases, 25.3%) and November (38 cases, 21.3%)(Table 2). The geographic analysis showed that 170 cases (95.5%) were from Ratlam district, while 8 cases (4.5%) were from neighbouring districts including Mandsaur, Ujjain, Dhar and Jhabua. Within Ratlam district, the majority of cases were from Ratlam city (urban areas), followed by various rural blocks (Table 3). The age-specific analysis revealed that the most affected age group was 21-30 years (29.2%), followed by 31-40 years (21.3%) and 11-20 years (18.0%). The paediatric population (≤10 years) accounted for 10.1% of cases. Gender-wise distribution showed male predominance across most age groups. Among the 178 cases, 118 (66.3%) were hospitalized (IPD) while 60 (33.7%) were managed as outpatients (OPD). The higher proportion of hospitalized cases suggests either severe disease presentation or the hospital's conservative approach to dengue management.

## Discussion:

This study provides comprehensive insights into the seroprevalence and epidemiological characteristics of dengue fever in north-western Madhya Pradesh. The overall seroprevalence of 9.38% among clinically suspected cases indicates a significant disease burden in the region and aligns with the national trend of increasing dengue incidence in India. The seroprevalence of 9.38% observed in our study is consistent with other hospital-based studies from central India. This rate reflects the efficiency of clinical screening protocols and the high specificity of NS1 antigen testing. The substantial number of positive cases (178 out of 1,898 tested) underscores the endemic nature of dengue in this region. The male predominance (63.5%) observed in our study is consistent with several previous studies from India [8, 9]. This gender distribution may reflect occupational exposure patterns, outdoor activities or healthcare-seeking behaviors rather than inherent susceptibility differences. The mean age of 28.1 years suggests that dengue primarily affects economically productive age groups, which has significant socio-economic implications. The highest incidence in the 21-30 years age group (29.2%) aligns with global patterns where young adults are predominantly affected [10]. This age group's higher mobility and outdoor exposure may contribute to increased risk of Aedes mosquito bites. The relatively lower incidence in children (10.1% in ≤10 year's group) may indicate either lower exposure or differences in healthcare-seeking behavior for pediatric patients. The distinct seasonal pattern observed in our study, with peak transmission during monsoon and post-monsoon months (August-November), is consistent with the ecology of Aedes mosquitoes. The remarkable peak in October (29.8% of cases) corresponds to the post-monsoon period when optimal temperature and humidity conditions, combined with accumulated water sources, provide ideal breeding conditions for Aedes aegypti and Aedes albopictus[11]. The extended transmission period from August to November (78.1% of all cases) indicates sustained vector activity and suggests the need for prolonged vector control measures. This temporal pattern emphasizes the importance of pre-monsoon vector control activities and community awareness programs. The concentration of cases in Ratlam district (95.5%) reflects both the hospital's catchment area and the urban nature of dengue transmission. Urban areas provide ideal conditions for Aedes mosquito breeding due to inadequate water storage practices, poor sanitation and high population density[12]. The limited cases from neighbouring districts suggest either lower disease burden in rural areas or patients seeking care at local facilities. The higher proportion of hospitalized cases (66.3%) compared to outpatients may indicate either severe disease presentation or the hospital's conservative approach to dengue management. This finding differs from some studies where outpatient management is more common [3]. The high hospitalization rate could also reflect the tertiary care nature of the hospital, which may receive more severe cases referred from primary and secondary healthcare facilities. The use of NS1 antigen detection proved highly effective for early dengue diagnosis. NS1 antigen detection has several advantages over other diagnostic methods, including early detection capability, high sensitivity and specificity and suitability for resource-limited settings[13].

## Public health implications:

The findings of this study have several important public health implications:

[1] Seasonal preparedness: The clear seasonal pattern necessitates enhanced surveillance and vector control activities before and during monsoon seasons, with particular focus on the August-November period.

[2] Targeted prevention: The demographic patterns suggest the need for targeted prevention strategies focusing on young adults and urban populations, particularly males in the productive age group.

[3] Healthcare planning: The high hospitalization rate indicates the need for adequate healthcare infrastructure and trained personnel during dengue seasons, with capacity building for managing severe cases.

[4] Community awareness: The geographic concentration of cases highlights the importance of urban-focused community awareness programs, emphasizing vector control and early diagnosis.

## Conclusion:

We show a significant burden of dengue fever in north-western Madhya Pradesh, with a seroprevalence of 9.38% among clinically suspected cases. The distinct seasonal pattern with peak transmission during August-November, male predominance and concentration in young adults provide important epidemiological insights for public health planning. Thus, we show the need for Enhanced pre-monsoon and monsoon vector control activities targeting urban areas with focus on water storage practices and breeding site elimination.

## Limitations:

The study is limited to a single tertiary care hospital, which may not represent the true community prevalence of dengue infection. Only clinically suspected cases were tested, potentially missing asymptomatic or mild cases that do not seek medical attention. While the study covers a complete calendar year, year-to-year variations in dengue transmission patterns were not assessed. The study did not include viral serotyping.

## Figures and Tables

**Table 1:** Demographic characteristics of dengue cases (n=178)

	**Category**	**Number (n)**	**Percentage (%)**
Gender	Male	113	63.5
	Female	65	36.5
Age Groups	≤10 years	18	10.1
	11-20 years	32	18
	21-30 years	52	29.2
	31-40 years	38	21.3
	41-50 years	21	11.8
	>50 years	17	9.6
Patient Type	IPD	118	66.3
	OP	60	33.7

**Table 2:** Monthly distribution of dengue cases

**Month**	**Number of Cases (n)**	**Percentage (%)**
January	2	1.1
February	0	0
March	1	0.6
April	1	0.6
May	3	1.7
June	0	0
July	5	2.8
August	22	12.4
September	45	25.3
October	53	29.8
November	38	21.3
December	8	4.5
Total	178	100

**Table 3:** District-wise distribution of dengue cases

	**Number of Cases (n)**	**Percentage (%)**
Ratlam	170	95.5
Mandsaur	3	1.7
Ujjain	4	2.2
Jhabua	1	0.6
Total	178	100
